# Activation of the NGF/TrkA signaling pathway attenuates diabetic erectile dysfunction

**DOI:** 10.18632/oncotarget.22389

**Published:** 2017-11-11

**Authors:** Yi Hou, Linpei Jia, Ying Zhang, Wei Ji, Hai Li

**Affiliations:** ^1^ Department of Urology, China-Japan Union Hospital of Jilin University, Changchun 130033, P.R. China; ^2^ Department of Nephrology, Xuanwu Hospital, Capital Medical University, Beijing 100000, P.R. China; ^3^ Department of Neurology, First Hospital of Jilin University, Changchun 130021, P.R. China; ^4^ Department of Vascular Surgery, Jilin Provincial People's Hospital, Changchun 130000, P.R. China

**Keywords:** the NGF/TrkA signaling pathway, diabetes mellitus, erectile dysfunction

## Abstract

Erectile dysfunction (ED) is a common complication of diabetes mellitus (DM). The exact role of the NGF/TrkA signaling pathway in the pathogenesis of diabetic ED is largely unknown. In the present study, we investigated the role of the NGF/TrkA signaling pathway in Sprague-Dawley rats with diabetic ED. Animals were divided into 2 groups: the normal group and the DM ED model group. The model group included the blank subgroup, the negative control (NC) subgroup, the TrkA subgroup and the TrkA + NGF subgroup. Erectile function, intracavernous pressure (ICP) and mean arterial pressure were measured respectively. Immunohistochemistry was used to examine the number of neuronal nitric oxide synthase (nNOS) expressing nerve fibers. The quantitative real-time polymerase chain reaction was applied to detect the mRNA expressions of NGF and TrkA in the cavernous tissue. Further, Western blotting was conducted to detect the expressions of NGF, TrkA and its downstream ERK pathway-related proteins. Higher erectile frequency, ICP values and diastolic function, more nNOS-positive nerve fibers, and decreased systolic function of the corpus cavernosum smooth muscle were found in the TrkA and TrkA+NGF groups when compared with the blank and the NC groups. Moreover, significantly higher mRNA expressions of NGF and TrkA, and upregulated protein expressions of NGF, TrkA, c-raf, ERK1/2 and CREB1 were found in the TrkA and the TrkA + NGF groups. In conclusion, downregulation in the NGF/TrkA signaling pathway may contribute to the pathogenesis of diabetic ED.

## INTRODUCTION

Erectile dysfunction (ED) is characterized by the inability to achieve or maintain an erection sufficient for perfect performance during sexual practice [1]. The most important organic causes of ED are vascular, diabetes, neurogenic, hormonal and side effects of drugs [2]. Epidemiological data have revealed that 35%-90% of diabetic men may suffer from ED [3, 4]. Moreover, the occurrence of ED is increased with age and the severity of diabetes mellitus (DM) [1]. As to the diagnosis of ED, the RigiScan device is the most reliable tool to diagnose ED and to distinguish psychogenic from organic cases by detecting penile tumescence and rigidity [5]. Common therapies of ED include oral phosphodiesterase-5 (PDE-5) inhibitors, vacuum constriction devices, intraurethral suppositories, hormones, intracavernosal injection of vasoactive agents, and prosthesis implantation [6]. However, the efficacy of the first-line medicinal treatment, namely PDE-5 inhibitors, is only about 51%-62% [7]. Therefore, it is urgent to search for more effective therapies to deal with diabetic ED.

Nerve growth factor (NGF) is seen as chemoattractant, which may regulate the proliferation and differentiation of cells and myelination of neurons. Besides, NGF functions on the cardiovascular system by binding with its known high-affinity receptor-TrkA. Blocking the NGF signaling reduced the neural invasion potential of pancreatic cancer cells [8]. NGF is also a main mediator of chronic pain [9]. TrkA is a member of the family of Trk and a cell surface transmembrane receptor kinase for NGF [10]. NGF and TrkA were discovered as neurotrophic factors crucial to facilitate the survival and the innervation of autonomic nerves and sensory neurons [11]. NGF plays an important part in the pathomechanism of diabetic polyneuropathy and NGF has therapeutic feasibility for cardiomyopathy in diabetic subjects [12, 13]. The NGF/tropomyosin-receptor kinase A (TrkA) signaling pathway is involved in regulating the state of vascular smooth muscle cells and cardiomyocytes [14]. The effectiveness of human adipose-derived stem cells and NGF-incorporated hyaluronic acid-based hydrogel application on the cavernous nerve (CN) has been reported [15]. Taken together, we hypothesize that the NGF/TrkA signaling pathway contributes to the erectile function and a downregulation in this signaling pathways may contribute to the pathogenesis of diabetic ED. In this regard, we aim to investigate the role of the NGF/TrkA signaling pathway in diabetic ED.

## RESULTS

### Successful establishment of the rat DMED model

At the 8^th^ week, the diabetic ED model was successfully established. One week after the establishment of animal model, fasting glucose of rats in the blank, the NC, the TrkA and the TrkA + NGF groups was significantly increased as compared with the normal group (*P* < 0.05). However, the values of fasting glucose among the blank, the NC, the TrkA and the TrkA + NGF groups were insignificantly different (*P* > 0.05, Table 1).

**Table 1:** Comparisons of blood glucose levels of rats among five groups (mmol/L)

Groups	First week	Second week	Third week	Fourth week	Fifth week	Sixth week	Seventh week	Eighth week
Normal	4.55 ± 1.67	4.30 ± 1.41	4.98 ± 1.88	4.67 ± 0.81	4.73 ± 0.90	5.32 ± 1.65	4.51 ± 0.89	5.10 ± 1.24
Blank	13.31 ± 1.30^*^	13.49 ± 1.35^*^	14.02 ± 0.91^*^	14.13 ± 1.38^*^	13.87 ± 1.21^*^	14.35 ± 0.92^*^	14.52 ± 1.12^*^	14.64 ± 1.23^*^
NC	12.85 ± 1.12^*^	13.14 ± 1.33^*^	13.87 ± 0.91^*^	13.52 ± 1.26^*^	13.41 ± 1.02^*^	13.77 ± 0.98^*^	13.89 ± 1.21^*^	14.10 ± 1.22^*^
TrkA	12.90 ± 1.12^*^	12.99 ± 0.89^*^	13.68 ± 1.21^*^	14.06 ± 1.32^*^	13.16 ± 1.02^*^	13.63 ± 1.41^*^	13.90 ± 1.24^*^	13.89 ± 1.21^*^
TrkA + NGF	12.88 ± 1.21^*^	12.98 ± 0.90^*^	13.55 ± 1.26^*^	13.68 ± 1.24^*^	13.22 ± 1.13^*^	13.72 ± 1.04^*^	14.02 ± 1.22^*^	13.97 ± 1.24^*^

Note: NC: negative control. ^*^P < 0.05, compared with the normal group.

Finally, 40 diabetic ED rats were divided into 4 groups: the blank group (n = 9), the NC group (n = 10), the TrkA group (n = 11) and the TrkA + NGF group (n = 10). In the four groups, all rats presented with symptoms including increased volume of drinking, feeding and urine, and decreased weight and activities.

### TrkA enhanced the erectile function in diabetic rats

**Figure 1:**
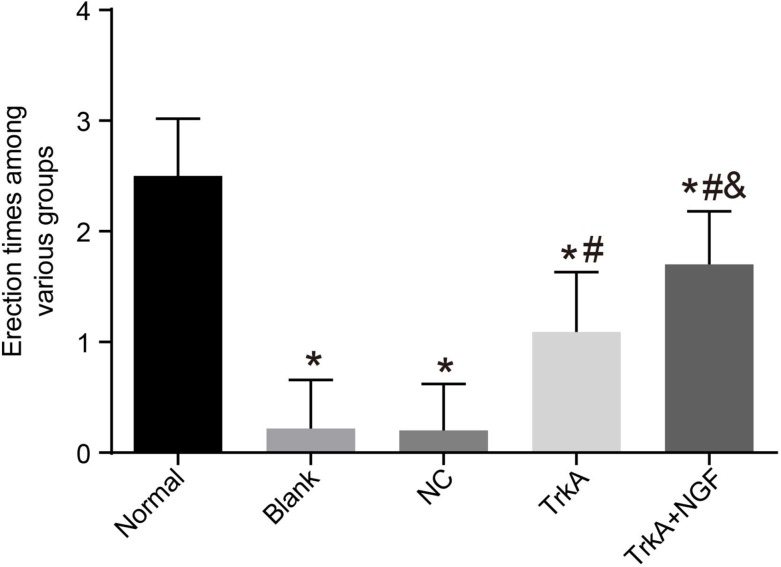
Erectile frequency of rats The times of penile erection of rats in the blank, the NC, the TrkA and the TrkA + NGF groups were significantly less than in the normal group (P < 0.05). Insignificant difference was found in comparisons of times of penile erection between the blank and the NC groups (P > 0.05). Compared with the blank and the NC groups, the times of penile erection of rats in the TrkA and the TrkA + NGF groups were increased, and compared with the TrkA group, the times of penile erection of rats in the TrkA + NGF group were increased (all P < 0.05). Note: NC: negative control. ^*^P < 0.05, compared with the normal group; ^#^*P* < 0.05, compared with the blank group; ^&^*P* < 0.05, compared with the TrkA group.

After injection of APO, rats in each group were characterized by yawning, restlessness, back of the foreskin, pushing the pelvis and erection of the penis. Times of penile erection of rats in the blank, the NC, the TrkA and the TrkA + NGF groups were less than that of the normal group (*P* < 0.05). No significant difference was found in comparisons of times of penile erection of rats in the blank and the NC groups (*P* > 0.05). Compared with the blank and the NC groups, times of penile erection of rats in the TrkA and the TrkA + NGF groups were increased, and compared with the TrkA group, times of penile erection of rats in the TrkA + NGF group were significantly increased (all *P* < 0.05, See Figure 1).

### TrkA increased the ICP values and NGF enhanced TrkA's effect

**Figure 2:**
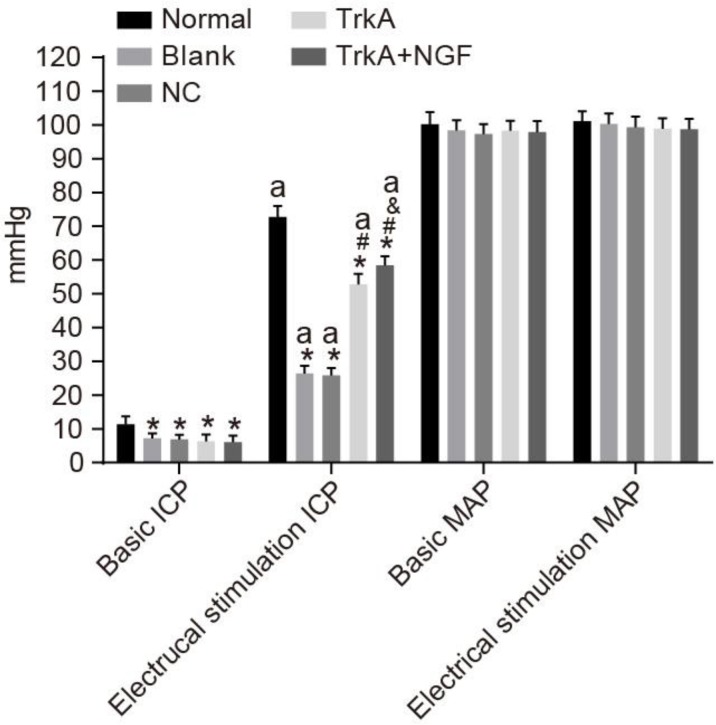
Comparisons of ICP and MAP of rats Compared with the normal group (the basic ICP [11.37 ± 2.41 mmHg]), the basic ICP of the blank, NC, TrkA and TrkA + NGF groups were decreased (*P* < 0.05). After electrical stimulation, MPG and ICP were increased in the normal group. In all groups, the ICP values after electrical stimulation and the basic ICP values were significantly different (*P* < 0.05). No significant difference was seen in the ICP values after electrical stimulation between the blank and the NC groups (*P* > 0.05). Compared with the blank and the NC groups, the ICP values after electrical stimulation were increased in the TrkA and the TrkA + NGF groups. When compared with the TrkA group, the ICP values after electrical stimulation were increased in the TrkA + NGF group (all *P* < 0.05). No significant difference of the basic MAP values was found among the groups (all *P* > 0.05). Note: ICP: intracavernous pressure; MAP: mean arterial pressure. ^*^P < 0.05, compared with the normal group;^#^*P* < 0.05, compared with the blank group; ^&^*P* < 0.05, compared with the TrkA group; ^a^*P* < 0.05, compared with the pre-electrical stimulation.

Compared with the normal group (the basic ICP value range: 11.37 ± 2.41 mmHg), the basic ICP values of the blank, the NC, the TrkA and the TrkA + NGF groups were decreased (all *P* < 0.05). After electrical stimulation, MPG and ICP were increased in the normal group. In all groups, the ICP values after electrical stimulation and the basic ICP values were significantly elevated (*P* < 0.05). No significant difference was seen in the ICP values after electrical stimulation between the blank and the NC groups (*P* > 0.05). Compared with the blank and the NC groups, the ICP values after electrical stimulation were increased in the TrkA and the TrkA + NGF groups. When compared with the TrkA group, the ICP values after electrical stimulation were increased in the TrkA + NGF group (all *P* < 0.05). No significant difference of the basic MAP values was found among groups (all *P* > 0.05, Figure 2).

### TrkA modulated the systolic/diastolic function of the corpus cavernosum smooth muscle

The systolic function of rats in the blank, the NC, the TrkA and the TrkA + NGF groups was superior to that of the normal group, but the diastolic function was inferior to that of the normal group (*P* < 0.05). The systolic and diastolic functions of rats were insignificantly different between the blank and NC groups (both *P* > 0.05). The systolic function of corpus cavernosum smooth muscle in the TrkA and TrkA + NGF groups was inferior (the TrkA group > the TrkA + NGF group) to, while the diastolic function was superior (the TrkA group < the TrkA + NGF group) to the blank and the NC groups (all *P* < 0.05) (Table 2).

**Table 2:** Comparison between diastolic and systolic function of corpus cavernosum smooth muscle in rats in each group

Group	Number (n)	Systolic function (%)	Diastolic function (%)
Normal	12	8.07 ± 2.52	40.51 ± 3.24
Blank	9	26.99 ± 2.82^*^	21.13 ± 2.11^*^
NC	10	27.99 ± 2.22^*^	20.96 ± 2.05^*^
TrkA	11	19.07 ± 2.21^*#^	27.93 ± 2.91^*#^
TrkA+NGF	10	15.74 ± 2.32^*#&^	35.57 ± 2.53^*#&^

Note: NC: negative control. ^*^P < 0.05, compared with the normal group; ^#^*P* < 0.05, compared with the blank group; ^&^*P* < 0.05, compared with the TrkA group.

### TrkA increased the number of nNOS-positive nerve fibers

**Figure 3:**
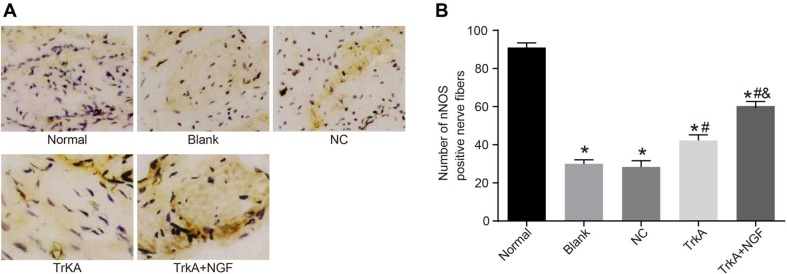
Immunohistochemical results of penile cavernous tissue in rats (× 400) and the number of nNOS positive nerve fibers In the normal group, the positive nerve fibers in the corpus cavernosum smooth muscle were evenly distributed, with clear staining and a large number of fibers. The number of the positive nerve fibers in the corpus cavernosum smooth muscle and the staining intensity in the blank, the NC, the TrkA and the TrkA + NGF groups were lower than in the normal group (*P* < 0.05). No significant difference was found in immunohistochemical results between the blank and the NC groups (*P* > 0.05). The number of the nNOS-positive nerve fibers in the corpus cavernosum smooth muscle in the TrkA and the TrkA + NGF groups was larger than in the blank and the NC groups. The number of the nNOS-positive nerve fibers in the corpus cavernosum smooth muscle in the TrkA + NGF group was larger than in the TrkA group (*P* < 0.05). Note: nNOS: neuronal nitric oxide synthase; NC: negative control. **(A)** Immunohistochemical staining of nNOS positive nerve fibers in the corpus cavernosum of rats. **(B)** Statistical analysis of nNOS positive nerve fibers in the corpus cavernosum of rats. ^*^P < 0.05, compared with the normal group; ^#^*P* < 0.05, compared with the blank group; ^&^*P* < 0.05, compared with the TrkA group.

The positive expression of nNOS was mainly in the small vessels and smooth muscle of the corpus cavernosum, and also around the dorsal penile nerve and urethral epithelium. In the normal group, the positive nerve fibers in the corpus cavernosum smooth muscle were evenly distributed, with clear staining, a large number of fibers (Figure 3). The number of the positive nerve fibers in the corpus cavernosum smooth muscle and the staining intensity in the blank, NC, TrkA and TrkA + NGF groups were lower than that of the normal group (*P* < 0.05). No significant difference was found in immunohistochemical results between the blank and the NC groups (*P* > 0.05). The number of the nNOS-positive nerve fibers in the corpus cavernosum smooth muscle in the TrkA and TrkA + NGF groups was larger than that of the blank and the NC groups. The number of the nNOS-positive nerve fibers in the corpus cavernosum smooth muscle in the TrkA + NGF group was larger than that of the TrkA group (*P* < 0.05).

### TrkA upregulated the mRNA expressions of NGF and TrkA in rat penile corpus cavernosum

**Figure 4:**
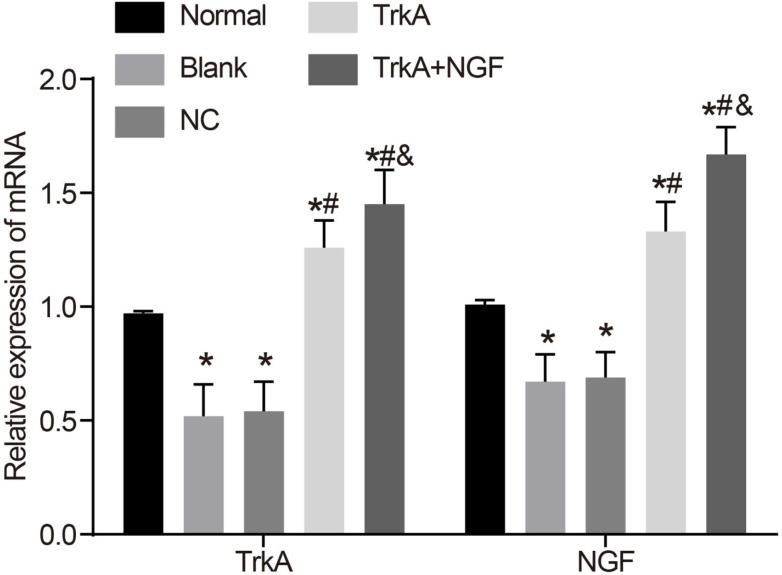
mRNA expressions of NGF and TrkA in rat penile corpus cavernosum Compared with the normal group, the mRNA expressions of NGF and TrkA in the blank and the NC groups were decreased (*P* < 0.05), while there was no significant difference in the mRNA expressions of NGF and TrkA between the blank and the NC groups (*P* > 0.05). The mRNA expressions of NGF and TrkA in the TrkA and the TrkA + NGF groups were higher than in the blank and the NC groups. The mRNA expressions of NGF and TrkA in the TrkA + NGF group were higher than in the TrkA group (*P* < 0.05). Note: NC: negative control. ^*^P < 0.05, compared with the normal group; ^#^*P* < 0.05, compared with the blank group; ^&^*P* < 0.05, compared with the TrkA group.

Compared with the normal group, the mRNA expressions of NGF and TrkA in the blank and NC groups decreased (*P* < 0.05), but there are no significant difference between the mRNA expressions of NGF and TrkA in the blank and NC groups (*P* > 0.05). The mRNA expressions of NGF and TrkA in the TrkA and the TrkA + NGF groups were higher than in the blank and NC groups. The mRNA expressions of NGF and TrkA in the TrkA + NGF group were higher than in the TrkA group (*P* < 0.05, Figure 4).

### TrkA upregulated the expressions of the NGF/TrkA signaling pathway-related proteins

**Figure 5:**
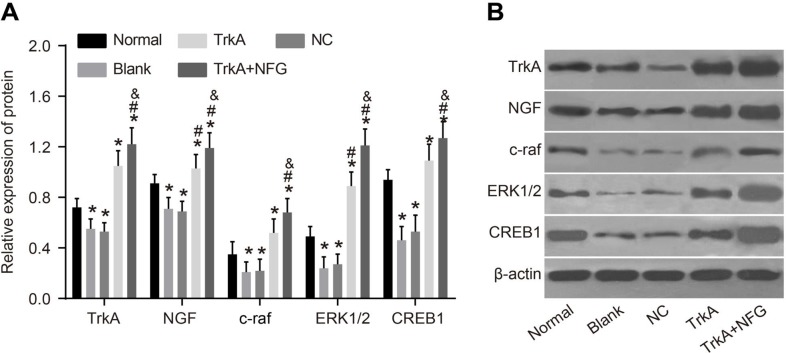
Protein expression of NGF/TrkA signaling pathway-related proteins in rat penile corpus cavernosum The Western blotting results are shown **(B)**. Further quantitative analysis by using the relative expression of protein **(A)** showed that compared with the normal group, the protein expressions of NGF, TrkA, c-raf, ERK1/2 and CREB1 in the blank and the NC groups were decreased (*P* < 0.05). Compared with the blank and the NC groups, the protein expressions of NGF, TrkA, c-raf, ERK1/2 and CREB1 in the TrkA and the TrkA + NGF groups were increased. Compared with the TrkA group, the protein expressions of NGF, TrkA, c-raf, ERK1/2 and CREB1 in the TrkA + NGF group were also increased (*P* < 0.05). Note: NC: negative control.^*^P < 0.05, compared with the normal group; ^#^*P* < 0.05, compared with the blank group; ^&^*P* < 0.05, compared with the TrkA group.

The NGF/TrkA signaling pathway-related proteins including NGF, TrkA, c-raf, ERK1/2 and CREB1 in the rat penile corpus cavernosum were measured by Western blotting. Compared with the normal group, the protein expressions of NGF, TrkA, c-raf, ERK1/2 and CREB1 in the blank and the NC groups were decreased (*P* < 0.05). Compared with the blank and the NC groups, the protein expressions of NGF, TrkA, c-raf, ERK1/2 and CREB1 in the TrkA and TrkA + NGF groups were significantly increased. Compared with the TrkA group, the protein expressions of NGF, TrkA, c-raf, ERK1/2 and CREB1 in the TrkA + NGF group were increased (*P* < 0.05, Figure 5).

## DISCUSSION

ED afflicts over one third of men throughout the world and exerts a negative influence on patients’ quality of life, psychosocial health, intimate relationships and self-esteem [18]. In this study, we explored the role of the NGF/TrkA signaling pathway in diabetic ED. Our results indicated that the activation of the NGF/TrkA signaling pathway improved the erectile function of rats with diabetic ED.

The erectile frequency and the ICP values after electrical stimulation in the TrkA and the TrkA + NGF groups were significantly increased (the TrkA group < the TrkA + NGF group). Our results are consistent with previous findings that NGF could restore erectile function in rats [19]. Nitric oxide synthase initiates cavernous tissue and vascular relaxation and endothelial nitric oxide synthase (eNOS) further facilitates blood flow into erectile tissue and maintains erection [15]. NGF can monitor NOS expression and NO production, and NO can modulate NGF-mediated neurotrophic responses [20]. Strong evidence indicated in the penile tissue, propofol's action may be due to increased synthesis of NO, the principal mediator of penile erection [21]. NGF up-regulates the expression of NOS isozymes which results in increased production of NO [22]. The ICP values were increased in NGF-treated diabetic animals [19], pointing to ICP as an important indicator of erectile function. In this regard, NGF may increase ICP via promoting the production of NO, whereby contributing to penile erection. Therefore, erectile function may be assessed by the ratio between ICP and MAP after electrical stimulation of cavernous nerve [23].

NO is a key mediator in the relaxation of cavernosal smooth muscle [24]. The constriction of corpus cavernosum smooth muscle is due to the lack of NO, and a balance between the production of relaxation and constriction is critical in maintaining the erectile function, so there is a negative correlation between diastolic and systolic function [25]. In a previous study, decreased contractile function of the heart was observed in the db/db diabetic mouse model [26]. We found that the systolic function of the corpus cavernosum smooth muscle was inferior (the TrkA group > the TrkA + NGF group) to the blank and the NC groups, while the diastolic function was superior (the TrkA group < the TrkA + NGF group) in the TrkA and the TrkA + NGF groups. In other words, NGF supplement leads to a decrease of systolic function and an increase of diastolic function in the corpus cavernosum smooth muscle. NGF might modulate NO production quickly and TrkA may function in a synergistic way.

Furthermore, the protein expressions of NGF, TrkA, c-raf, ERK1/2 and CREB1 in the TrkA and the TrkA + NGF groups were increased (the TrkA group < the TrkA + NGF groups). Consistent with our results, Liu et al. revealed that after epigallocatechin-3-gallate treatment, TrkA signaling was sparked off by raising the phosphorylation of TrkA following the enhanced phosphorylation of c-Raf, ERK1/2, and CREB [27]. NGF can increase a small amount of secretion to boost the recovery of nerve injury and activate ERK mitogen-activated protein kinases, which trigger off the pp90 ribosomal S6 kinase family of Ser/Thr kinases in turn, all members of which facilitate CREB Ser-133 phosphorylation [28]. NGF plays a part in the time course of endogenous c-Raf activation in mammalian cells [29]. Combination of NGF to TrkA triggered off protein kinases and transcription factors [23], and subsequently, the protein expressions of NGF, TrkA, c-raf, ERK1/2 and CREB1 in the TrkA and TrkA + NGF groups were increased.

In summary, the activation of NGF/TrkA signaling pathway improves the erectile function of rats with diabetes mellitus, which suggests a promising perspective in the future clinical practice.

## MATERIALS AND METHODS

The study protocol was approved by the Committee on the Ethics of China-Japan Union Hospital of Jilin University. All efforts were made to minimize the number of animals used and their sufferings.

### Animals

A total of 60 male Sprague-Dawlay (SD) rats (20-week-old) with a weight range of 200 g to 250 g at clean grade were used. All rats were purchased from Slac laboratory animal company (Shanghai, China. Animal License No: SCXK (Shanghai) 2003-0003) and were housed on a 12/12 light-dark schedule with water and food available *ad libitum*. Detailed accommodation and care complied with Chinese recommendations and legislations. The mating test showed that the sexual function of all rats was normal.

### Establishment of diabetic rat model

A total of 48 rats were chosen to establish rat diabetic model [16]: 10 g/L fresh streptozotocin (STZ) solution was made up by 0.1 mol/L sodium citrate buffer (pH = 4) in 4°C water-bath (the solution was prepared when needed). All rats were immediately injected (STZ 60 mg/kg) via the left lower abdominal cavity after 12 hours’ fasting, and 72 hours later, blood glucose values from the tail vein were measured by the Johnson glucose meter. Models were successfully established when the glucose level exceeds 16.6 mol/L.

### Selection of ED rat model

The rat ED model was made according to theHealton's method [17]. At the 8^th^ week after the establishment of diabetic model, rats were weighed and then put into observation boxes. After 10 minutes adaptation to the environment, rats were observed in the dark and quiet room. After subcutaneous injection of apomorphine (80 μg/kg, APO, Sigma Company, USA) via the neck, rats were observed again for 30 minutes. Rats showed the pelvis forward and glans penis and terminal penis exposed under upright posture was regarded as penile erection.

### Treatment

SD rats were divided into 2 groups: the normal group (n = 12) and the model group (n = 48); the model group was sub-divided into the blank group, the negative control (NC) group, the TrkA group (injection of TrkA expression vector) and the TrkA + NGF group (injection of TrkA expression vector and hNGF). In the normal and blank groups, rats were injected with 12 IU/kg sodium citrate buffer via peritoneal injection, once a night. In the NC group, rats were injected with 12 IU/kg pBPSTR1 empty plasmid carrier solution (National Institutes of health, Bethesda, Maryland, USA) via tail vein, and the same volume of sodium citrate buffer via peritoneal injection once a night. In the TrkA group, rats were injected with 12 IU/kg pBPSTR1-TrkA empty plasmid carrier solution (National Institutes of health, Bethesda, Maryland, USA) via tail vein, and the same volume of sodium citrate buffer via peritoneal injection, once a night. In the TrkA+NGF group, rats were injected with 12 IU/kg pBPSTR1-TrkA empty plasmid carrier solution via tail vein, and 250 IU/kg hNGF (Military Science Research Institute of Nanjing military region of PLA, Nanjing, Jiangsu, China) via peritoneal injection, once a night. The administration time lasted 8 weeks.

### Experiments on intracavernous pressure (ICP) and electric stimulation of cavernous nerve

Diabetic animals with ED were selected and ICP was measured after 8 weeks administration. Rats were anaesthetized by injection of 10 mg/kg Ketamine via peritoneal injection, and carotid artery was cut in the middle necks, followed by the connection of cannula with transducer and constantly monitor of mean arterial pressure (MAP). Median incision of lower abdomen was selected for observation of posterior surface of prostate, and finding that cavernous nerve and major pelvic ganglion (MPG) were revealed. After full exposure of penis and bulbocavernosus muscle, a total of 250 U/mL heparin solution and syringe needle of trocar linking with catheters were respectively inserted into the both corpus cavernosum about 5 mm deep at the edge of glans penis and somatic junction via paralleling with scapus penis, and well fixed. After that, transducer was linked with several physiological recorders to monitor ICP. A bipolar stainless-steel hook electrode, linking with the output terminal of a computer, was used to stimulate MPG (parameter of stimulation was 5 V, 5 ms, 12 Hz), lasting 30 s, and pressure change data were collected and analyzed.

### Detection of systolic and diastolic function of penile corpus cavernosum smooth muscle

After anesthesia, penis was cut off. Samples were collected and kept with 4°C Krebs solution. Corpus cavernosum penis was exposed and made into a muscle strip in 6 mm X 2 mm X 2 mm, and one end was fixed in a thermostatic bath, and the other end was linked with a tension transducer. When the muscle strip balanced, tension of the smooth muscle F2 was recorded, and then tension of the smooth muscle F1 was recorded again after addition of 50 μmol/L PE. (F1-F2)/F1 got contraction percentage. Krebs solution was used to remove the effect of PE; tension F3 was recorded after adding Ach 100 μmol/L. (F1-F3)/F1 represented relaxation percentage.

### Immunohistochemical detection of nNOS

Samples were collected after measuring ICP of rats in all groups. Lower margin of pubic symphysis was cut off from the root of the penis, fascia and prepuce were removed and 10% neutral formaldehyde solution was added to fix glans penis. Samples were embedded in paraffin and cut into sections with 4 μm thickness, dehydrated by 75%-100% alcohol and vitrificated by dimethylbenzene for 30 minutes. Wax dipping, embedding, and final sections (4 μm thickness) were conducted. Sections were conventional dewaxed by xylene solution, dehydrated by graded alcohol, and washed 3 times by distilled water. And then, sections were put into 0.01 mol/L citrate buffer (pH6.0) to repair antigen by high pressure method, then cooled and washed by distilled water and 0.01 mol/L PBS (phosphate buffer saline) (pH7.2) 3 times (3 minutes each time). Sections were immersed in 3% H_2_O_2_ (freely prepared) for 10 minutes and washed by PBS 3 times (3 minutes each time). Serum blocking solution of normal goats was added and kept at room temperature for 10 minutes and 50 μl rabbit polyclonal antibody nNOS (IgG diluted at 1:100, Neo Marker Company, USA) was also added for incubation at 4°C overnight, followed by PBS washing for 3 times (3 minutes each time). A second antibody of horse-radish peroxidase (HRP) labeled goat anti-rabbit IgG (50 μl) was added (Dako Company, USA, EnVision Method), incubated at room temperature for 20 minutes, washed by PBS for 3 times (3 minutes each time), and developed by DAB (Dako Company, USA) for 5-10 minutes. PBS, instead of the first antibody, was used as a negative control. After dehydration, vifrification and mounting, staining result was observed. The number of positive staining fibers under 5 high-power fields (HPF) was randomly calculated by each section. And 50 sections of images were randomly selected under 400 HPF, and the analysis system of MPIAS-2000 high definition color pathological image was used to analyze the images.

### qRT-PCR

The posterior tissues of fresh penile corpus cavernosum (about 200 mg) were selected and cut into pieces. Cells and RNA were collected and extracted according to the instruction for the Trizol kit (Invitrogen, Waltham, MA, USA). Total RNA from tissue samples were extracted by one-step method of Trizol. Ultra-pure water, processed by diethylpyrocarbonate (DEPC, Shanghai Sangon Biological Engineering Technology & Services Co., Ltd. Shanghai, China) was used to dissolve RNA, and ND-1000 UV/VIS spectrophotometer (Nanodrop Company, USA) was applied to measure absorbance at 260 nm and 280 nm. The quality of RNA was identified and the concentration was adjusted. According to the instruction for the reverse transcriptase kits (PrimeScript, Takara Company, Japan), a two-step method was used to finish reverse transcription. The reaction condition was: 70°C for 10 minutes, ice bath for 2 minutes, 42^°^C for 60 minutes, and 70°C for 10 minutes. cDNA obtained by reverse transcription were temporarily stored at −80°C. Real-time PCR was applied to detect the mRNA expression of NGF and TrkA, and 1 μl cDNA was selected for PCR reaction. The reaction condition was: pre-denaturation at 94°C for 5 minutes, denaturation at 94°C for 30 seconds, annealing at 60°C for 30 seconds, and extension at 72°C for 30 seconds, for 40 circles, and the extension again at 72°C for 10 minutes. PCR reaction was finished by ABI 7500 detecting system, and raw data were collected and analyzed by SDS 7500. The β-actin gene was selected as a reference gene, and each gene in the samples had a corresponding Ct (cycle threshold) value. The data were analyzed by 2^−ΔΔCt^, which indicated the ratio of gene expression between the experimental group and control group. The formula was: ΔΔCT = ΔCt_experiment group_ - ΔCt_control group_, and ΔCt = Ct_target gene_ - Ct_β-actin._ The expression changes of every gene were expressed by geometric mean. Each experiment was repeated twice at least. Primer sequence and fragment size of PCR products were shown in Table 3.

**Table 3:** Primer sequences for qRT-PCR

Gene	Primer sequences	Size
TrkA	F: 5′-TTTGAGTTCAACCCTGAGGACCCC-3′	523bp
	R: 5′-TCCCCTAGCTCCCACTTGAGAATG-3′	
NGF	F: 5′-GCCTCAAGCCAGTGAAATTAGG-3′	366bp
	R: 5′-ACGACCACAGGCCAAAACTC-3′	
β-actin	F: 5′-GATGGTGGGTATGGGTCAGAAAGGA-3′	229bp
	R: 5′-GCTCATTGCCGATAGTGATGACCG-3′	

Note: qRT-PCR: quantitative real-time polymerase chain reaction.

### Western blotting

After the corpora cavernosum penis was cut into pieces, RIPA lysis buffer was added. Protein was smashed by ultrasonic, pre-colded and centrifuged at 4°C, and then supernatant was extracted. The protein concentrations were measured by a BCA Kit (Beyotime Biotechnology, Shanghai, China), and the total volume of extracted protein was 50 μg, adding loading buffer and boiling at 100^°^C for 10 minutes. After SDS polyacrylamide gel electrophoresis (PAGE) (Boster, Hubei, Wuhan, China), target protein was transferred into polyvinylidene fluoride (PVDF) membrane, and then PVDF membrane was sealed in 5% bovine serum albumin (BSA) for 2 hours. After sealing, rabbit anti-mouse (RAM) NGF antibody (1:1000, abcam company), RAM TrkA antibody, RAM c-raf and ERK1/2 (1:1500, CST Company), RAM CREB1 antibody (1:500, Santa Cruz Biotechnology), and RAM β-actin (1:2000, Santa Cruz Biotechnology) were added into for incubating one night. The PVDF membrane was taken out and washed three times (10 minutes each time). A second antibody of HRP labeled-goat anti rabbit IgG (1:4000) was added according to the introduction, and incubated for 1 hour and washed three times again (10 minutes each time). A chemical luminescence reagent ECL (Beyotime Biotechnology, Shanghai, China) was applied to collect and process images, and the acquired images were analyzed by Quantity One.

### Statistics

Data were presented as mean values ± standard deviations (SD). The Statistical Program for Social Sciences (SPSS) 19.0 software (SPSS, IBM, West Grove, PA, USA) was used for data analysis. The one-way analysis of variance (ANOVA) and the Kruskal-Wallis test were used to compare values among groups followed by the Student's t-test or the Mann-Whitney u-test to compare values between groups. All tests were two-tailed, with the level of significance set to *p* < 0.05.
